# Recent trends of “manels”: gender representation among invited panelists at an international oncology conference

**DOI:** 10.1093/jncics/pkad008

**Published:** 2023-02-10

**Authors:** Sophia C Kamran, Beow Y Yeap, Anushka Ghosh, Christopher M Aldrighetti, Henning Willers, Neha Vapiwala

**Affiliations:** Department of Radiation Oncology, Massachusetts General Hospital, Harvard Medical School, Boston, MA, USA; Department of Medicine, Massachusetts General Hospital, Harvard Medical School, Boston, MA, USA; Department of Radiation Oncology, Massachusetts General Hospital, Harvard Medical School, Boston, MA, USA; Department of Radiation Oncology, Massachusetts General Hospital, Harvard Medical School, Boston, MA, USA; Department of Radiation Oncology, Massachusetts General Hospital, Harvard Medical School, Boston, MA, USA; Department of Radiation Oncology, Hospital of the University of Pennsylvania, Perelman School of Medicine, Philadelphia, PA, USA

## Abstract

**Background:**

Gender disparities in academic medicine are a long-acknowledged concern, particularly at medical conferences. We investigated gender representation and prevalence of “manels” (all-men panels) among invited speakers at the 2018-2021 American Society of Clinical Oncology Annual Meetings.

**Methods:**

Using American Society of Clinical Oncology online programs, 2018-2021 faculty information was obtained, including perceived or self-reported gender, medical specialty, session type, and topic. Primary outcomes were percentage of manels and proportion of women panelists over time; women representation among specialties and topics were evaluated. Cochran-Armitage and Fisher’s exact tests were used to analyze trends in proportion of manels and women representation over time and to compare each session type, topic, or specialty with other categories combined, respectively.

**Results:**

During 2018-2021, there were 670 sessions, 81 of which (12.1%) were manels. Among 2475 panelists, 1181 (47.7%) were women. Over time, the percentage of manels significantly decreased from 17.4% in 2018 to 9.9% in 2021 (*P* = .030). The highest proportion of manels was observed for leadership or special sessions (17.1%, *P* = .419). Women panelists were underrepresented for the topics of genitourinary cancers (38.6%, *P* = .029) and translational or preclinical sciences (36.7%, *P* < .001). There was a positive trend toward improved women representation among translational or preclinical sciences (27.4% in 2018 vs 41.8% in 2021, *P* = .031) but not among genitourinary cancers (41.1% in 2018 vs 40.7% in 2021, *P* = .969).

**Conclusions:**

The number of women panelists increased during the study period, with a corresponding decrease in the proportion of manels, specifically in education and leadership or special sessions. Ongoing underrepresentation of women in genitourinary cancers and translational or preclinical topics underscores the importance of annual meeting organizers continuing to strive for diverse gender representation.

In June 2019, National Institutes of Health (NIH) Director Francis S. Collins MD, PhD, publicly called for an end to all-men speaking panels (“manels”) (1). This was in response to the report by the National Academy of Sciences, Engineering, and Medicine released in 2018 that discussed discriminatory behavior toward women in the fields of science, engineering, and medicine and the extent to which it limits women’s careers (2). The report outlined multiple strategies to prevent and address this discriminatory behavior, notably the role of scientific leaders to change the culture of organizations. NIH Director Collins established an expectation of a more level playing field for speaking opportunities and challenged other scientific leaders across the biomedical field to do the same. Whether large academic oncology conferences followed suit has remained understudied.

In medical schools, gender parity has been achieved since the early 2000s, with more women than men matriculated since 2019 (3). However, women faculty are underrepresented in academic medicine, particularly at higher academic ranks and in leadership positions (4). In the oncology workforce, a recent report evaluating medical and radiation oncology academic faculty demonstrated lack of women in higher academic ranks and at academic chair levels, with women comprising only 21% of full professors in radiation oncology and 28% of full professors in medical oncology (5). It is well known that invitations to speak at major meetings provide visibility and are a key metric used by faculty promotion and tenure committees for professional advancement. National reputation is generally intertwined with academic achievement as well as self-promotion and outspokenness, the latter of which, stereotypically, men are better at than women. Thus, gender disparity at higher academic ranks may be at least partly related to gender imbalances among speaking and panel roles at high-profile professional meetings.

The American Society of Clinical Oncology (ASCO) Annual Meeting is one of the largest international oncology conferences, with more than 30 000 attendees yearly. It is also an opportunity for clinicians and scientists to network and connect with colleagues to form academic, educational, and research collaborations. Thus, we sought to investigate the prevalence of manels and gender representation of invited panelists at the ASCO Annual Meeting between 2018 and 2021.

## Methods

Using ASCO online programs, 2018-2021 sessions were reviewed. Faculty information was obtained for those who participated in a panel (defined as a session with minimum 2 speakers, including a chair or moderator); data were extracted by mixed-gender coders. Faculty and presenter information was not obtained for those who presented original research because scientific abstracts selected for presentation are based on merit, and abstract presenters can select alternates to present in their absence, whereas participation in a panel or as a panel chair or moderator is generally ASCO committee appointed. Data collected included perceived or self-reported gender that was based on the panelist’s institutional website or their professional website. Where possible, these were confirmed with the National Provider Identifier (NPI) database, where gender is a required field. Of note, the NPI database asks for “gender” with options of “male/female,” which is generally noted as a biological definition (not a gender identity). The NPI database only provides the options of “male” and “female”—there is no opportunity to select or input anything else. Thus, for the purposes of this analysis, gender was extracted as binary. Also collected were medical specialty, panel role (chair or moderator vs nonchair or nonmoderator), session type, and topic. For 2021 panelists, academic position (when available), number of publications, number of citations, and H-index were retrieved from Web of Science and Scopus between September and December 2021. The Mass General Brigham Institutional Review Board deemed this study as exempt from formal review because of use of public information.

Primary outcomes included percentage of manels (defined as panels comprised of all men) and proportion of women panelists. Representation of women among chair or moderator role, specialties, session type, and topic were evaluated. The gender distribution of individual panelists participating in more than 1 role was evaluated. Manel sessions were evaluated by session type and topic.

### Statistical analysis

The Cochran-Armitage test was used to analyze trends in the proportion of manels and representation of women over time. Fisher’s exact test was used to compare the gender distribution between each session type, topic, or specialty with other categories combined and across academic rank. For 2021, analysis was performed by unique panelist, and Wilcoxon rank-sum test was used to compare the number of publications, number of citations, and H-index between genders. *P* values are based on a 2-sided hypothesis. All statistical analyses were performed using SAS 9.4 (SAS Institute Inc, Cary, NC, USA).

## Results

**Figure 1.**
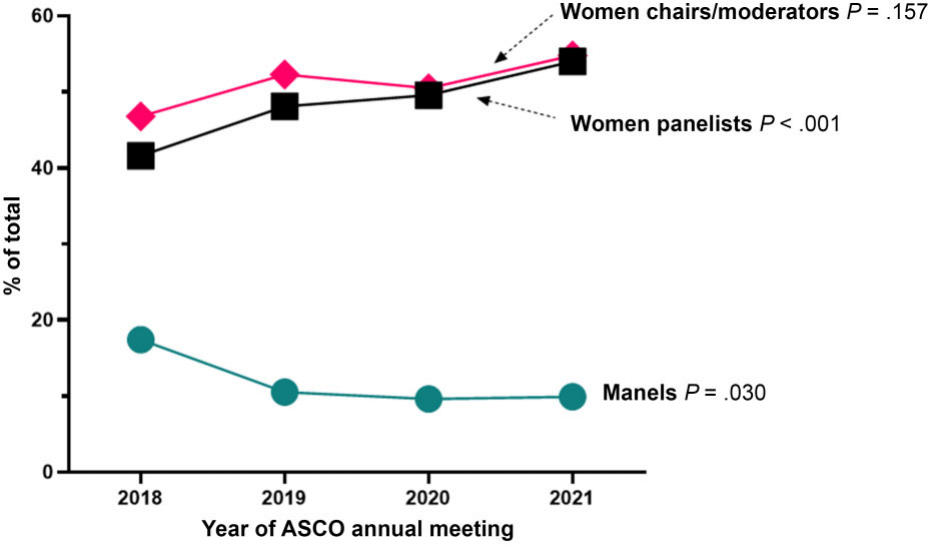
Percentage of women panelists, chairs or moderators, and manels at American Society of Clinical Oncology Annual Meetings. Between 2018 and 2021, there was a decrease in the percentage of manels, from 17.4% to 9.9% (*P* = .030) and a corresponding increase in the proportion of women panelists from 41.6% to 54.0% (*P* < .001). In addition, there was an increase in women chairs or moderators, from 46.8% in 2018 to 54.8% in 2021 (*P* = .157). The *y*-axis represents percent of total panels, panelists, or chairs or moderators, for each line graph, respectively.

During the ASCO Annual Meetings from 2018 to 2021, there were 670 panels total, 81 of which (12.1%) were manels. Among 2475 panelists, 1181 (47.7%) were women. Over time, there was a statistically significant decrease in the number of manels, from 17.4% (33 of 190 panels) in 2018 to 9.9% (15 of 151 panels) in 2021 (*P* = .030) and a corresponding increase in proportion of women panelists from 41.6% to 54.0% (*P* < .001) (Figure 1). In addition, the role of chair or moderator was a majority of men (53.2%) in 2018, but since 2019, women represent more than 50% of total chairs or moderators (52.3% in 2019, 50.5% in 2020, and 54.8% in 2021; *P*_trend_ = .157).

### Women panelist representation

**Figure 2.**
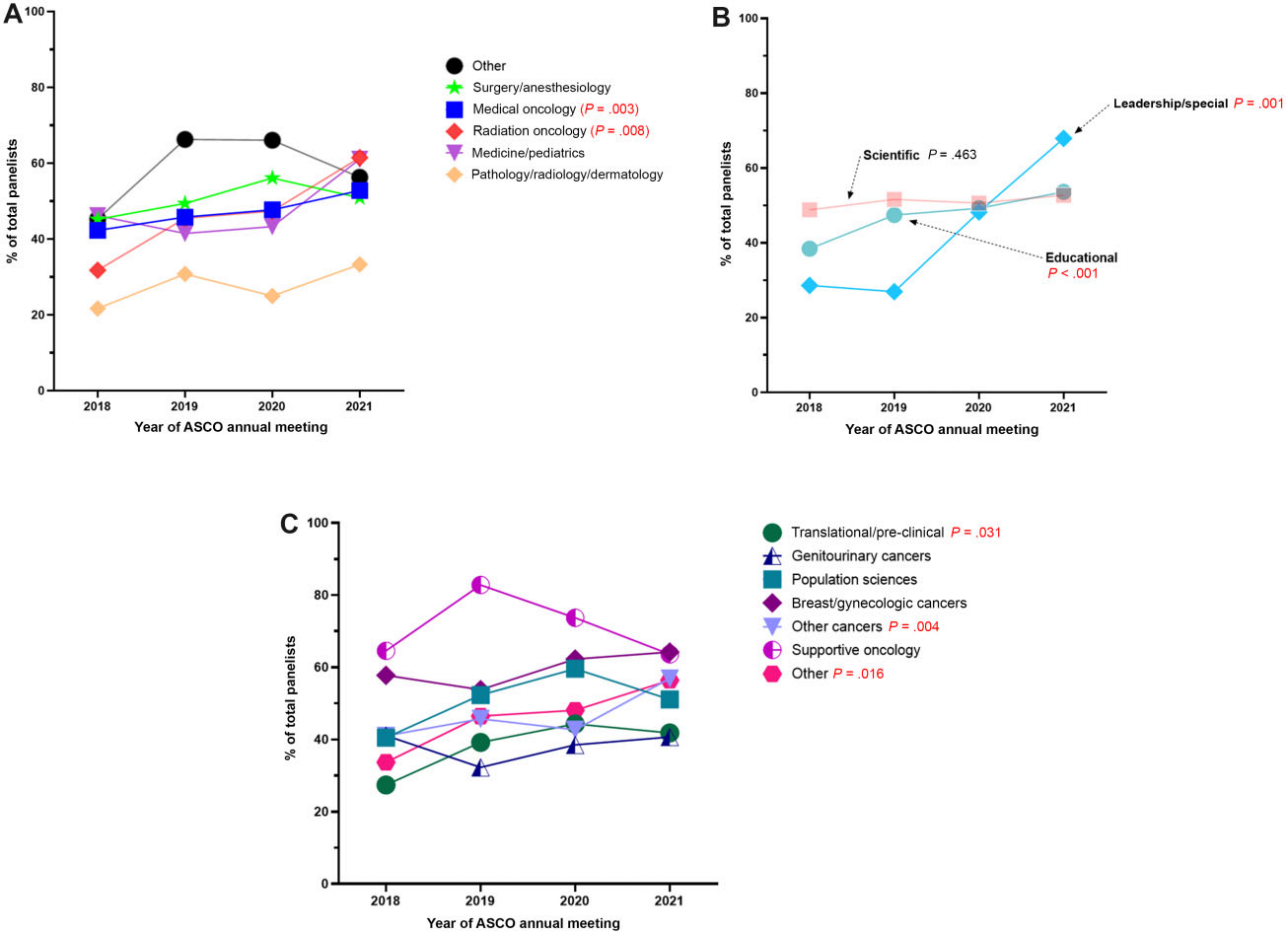
Percentage of women panelists by specialty, session type, and topic. **A**) By specialty, there was a significant increase in percentage of women panelists in medical and radiation oncology between 2018 and 2021, from 42.3% in 2018 to 52.8% in 2021 for medical oncology and 31.8% in 2018 to 61.5% in 2021 for radiation oncology. **B**) There was a significant increase in the percentage of women on leadership or special session panels (*P* = .001) and educational panels (*P* < .001) between 2018 and 2021, with no significant change in representation for scientific session panels (*P* = .463). **C**) There were improvements in representation of women in certain panel session topics, including translational or preclinical sciences, from 27.4% in 2018 to 41.8% in 2021 (*P* = .031). There were also positive trends in representation of women in the topics of other cancers (which include hematologic, gastrointestinal, lung, CNS, head and neck, pediatric, melanoma or skin, sarcoma) as well as “Other” topics. *Specialties*: Surgery: surgery, gynecology, otolaryngology, neurosurgery, general surgery, plastic surgery. Medicine: internal medicine, family medicine, pulmonary medicine, gastroenterology, cardiology, endocrinology. Radiology: radiology, nuclear medicine. Other: nonclinical, immunology, pharmacology, palliative care, psychiatry, geriatrics, other, unknown. *Topics*: Population sciences: care delivery and regulatory policy, disparities/health equity, prevention, risk reduction, hereditary cancer, health services and quality improvement, global health, ethics. Other cancers: hematologic, gastrointestinal, lung, CNS, head and neck, pediatric, melanoma or skin, sarcoma. Translational or preclinical: developmental therapeutics, immunotherapy, tumor biology, precision medicine, cancer genetics. Supportive oncology: symptoms and survivorship, patient and survivor care, geriatric. Other: special sessions, professional development and education advances, clinical trials, award lecture.

In terms of specialty representation, the representation of women panelists in medical oncology and radiation oncology statistically significantly increased over time, from 42.3% (224 of 530) in 2018 to 52.8% (167 of 316) in 2021 for medical oncology (*P* = .003) and 31.8% (14 of 44) in 2018 to 61.5% (24 of 39) in 2021 for radiation oncology (*P* = .008; Figure 2, A). The lowest proportions of women panelists were in pathology, radiology, and dermatology specialties (26.2%, *P* = .001; Supplementary Table 1, available online).

Representation of women on leadership or special session panels improved between 2018 and 2021, from 28.6% (8 of 28) in 2018 to 67.9% in 2021 (19 of 28, *P* = .001). Representation of women also improved for educational panels, from 38.4% (176 of 458) in 2018 to 53.6% (157 of 293) in 2021 (*P* < .001). There was no statistically significant change in representation on scientific panels over time (from 48.8% in 2018 to 52.7% in 2021; *P* = .463; Figure 2, B).

Women panelists were underrepresented for the topics of genitourinary cancers (38.6%, *P* = .029) and translational or preclinical sciences (36.7%, *P* < .001). However, there was a positive trend toward improved women representation among translational or preclinical sciences (27.4% in 2018 to 41.8% in 2021, *P* = .031). In contrast, there was no further improvement among genitourinary cancers (41.1% in 2018 to 40.7% in 2021; *P* = .969; Figure 2, C).

In evaluating individuals with more than 1 role (ie, someone who serves as a panelist in more than 1 panel), we found a substantial decrease overall over time from 7.6% (51 of 670) in 2018 to 3.2% (16 of 501) in 2021 (*P* < .001; Table 1). There were 8.2% (23 of 282) of women who served in more than 1 role in 2018, which reduced to 3.8% (10 of 266) in 2021 (*P* = .011), and there were 7.2% (28 of 388) of men who served in more than 1 role in 2018, which reduced dramatically to 2.6% (6 of 255) in 2021 (*P* = .005).

**Table 1.** Men and women with less than 1 role

Category	2018-2021		2018		2019		2020		2021		*P*_time trend_
	n > 1 role (%)	Total No.	n > 1 role (%)	Total No.	n > 1 role (%)	Total No.	n > 1 role (%)	Total No.	n > 1 role (%)	Total No.	
Women	57 (5.1)	1107	23 (8.2)	282	17 (5.2)	327	7 (3.0)	232	10 (3.8)	266	.011
Men	58 (4.8)	1214	28 (7.2)	388	16 (4.5)	353	8 (3.4)	238	6 (2.6)	235	.005
Women and men	115 (5.0)	2321	51 (7.6)	670	33 (4.9)	680	15 (3.2)	470	16 (3.2)	501	<.001
*P*_women vs men_		.702		.661		.724		>.99		.612	

### Manel sessions by type and topic

Among session type and topic, the highest proportion of manels was observed for leadership or special sessions (17.1% vs 12.1% manels overall, *P* = .419) and translational or preclinical topics (19.6% vs 12.1% manels overall, *P* = .024). The lowest proportion of manels was observed for scientific sessions (4.2%, *P* < .001) and supportive oncology (2.8%, *P* = .110; Supplementary Table 2, available online). The proportion of manels decreased over time among educational sessions from 22.2% in 2018 to 12.9% in 2021 (*P* = .037) and leadership or special sessions from 25.0% in 2018 to 0% in 2021 (*P* = .057). In contrast, there were some all-women panels, from 12 in 2018 (6.3%), 10 in 2029 (5.8%), 20 in 2020 (12.7%), and 19 in 2021 (12.6%).

### Comparison of men and women panelists in 2021

In 2021, although there were more women panelists (54%), men held a higher academic rank (43.4% vs 36.5% full professor, *P* = .028) and had a greater number of publications (median 116 vs 83.5, *P* < .001), citations (median 5321 vs 2657.5, *P* < .001), and higher H-index (median 33 vs 25, *P* < .001) than women (Table 2).

**Table 2.** Comparison between women and men panelists^a^ at ASCO 2021

Rank	Women	Men	*P*
	(n = 266)	(n = 235)	
Professor, No. (%)			
All	97 (36.5)	102 (43.4)	
Associate professor	61 (22.9)	48 (20.4)	
Assistant professor	56 (21.1)	34 (14.5)	.0282^b^
Other^c^	52 (19.6)	51 (21.7)	
Publications, median (interquartile range)			
All	83.5 (134)	116 (176)	<.001
Citations	2657.5 (8179.5)	5321 (10660)	<.001
H-index	25 (31)	33 (32)	<.001

a Including chairs. ASCO = American Society of Clinical Oncology.

b *P* value excluding “Other” category.

c Other: adjunct faculty, nonacademic appointment.

## Discussion

In the oncologic specialties, failure to achieve gender parity among academic faculty at the highest levels of rank or leadership has been demonstrated (5) despite the increasing number of women entering oncology but hovering at lower-rank levels. Scientific society annual meetings serve as important platforms for clinicians and scientists to build a national reputation, gain visibility, and network with collaborators for professional growth and research endeavors. Serving as an invited panelist is critical for promotion and tenure within academia. Overall, at the ASCO Annual Meetings between 2018 and 2021, we found that the proportion of women panelists increased over the study period from 41.6% to 54.0%, with a corresponding decrease in the proportion of manels from 17.4% to 9.9%, demonstrating an improvement in representation of women on panels. There was a striking improvement in women representation in leadership and special sessions as well as educational sessions, with associated decreases in the proportion of manels over time. However, we did find topic areas where there was continued underrepresentation of women (eg, genitourinary cancers and translational or preclinical topics). In addition, the percent of manels among all sessions has remained at approximately 10% since 2019, with no further improvement, even with virtual meetings in 2020 and 2021, due to the COVID-19, or SARS-CoV-2, pandemic. Although in 2018 it was common to have individuals from either gender serve in multiple panelist roles, thankfully in 2021 this trend was dramatically reduced among both men and women. This reduction creates additional speaking opportunities for others. We are encouraged by the improvement in diverse representation of voices among invited panelists at the ASCO Annual Meeting, yet it is imperative that ASCO committees, comprised primarily of volunteer clinicians and scientists from across the world, as well as ASCO leadership be aware of the areas with continued limited representation.

Achieving gender equity at the national level has been recognized as an important goal for many different fields. The Lancet announced a No All-Male Panel Policy for Lancet Group editors as part of their commitment to increasing gender equity, diversity, and inclusion in scientific research and publishing (6). There has been a large social media movement against manels with the Twitter handle @ManelWatchUS as well as others for manels globally. Other fields have started the process of critical introspection to understand their current progress toward achieving gender parity among speakers at large annual conferences. The importance of identifying the prevalence of manels within a field or society cannot be underscored because it is often unintentional and a function of “the first name that comes to mind,” but deliberate efforts can effect substantial change. The American Society for Microbiology Annual Meeting speakers were analyzed between 2011 and 2013, and it was found that an average of 29.6% speakers were women for the 3 years combined (7). These findings were presented to the leadership and program planning committee for the 2014 meeting, with subsequent analysis demonstrating increased representation of women to 43% at that meeting. These findings were again presented to the 2015 planning committee, with specific instruction to “do better” with respect to gender balance and to avoid sessions with all men, except under “extraordinary circumstances.” The society achieved close to gender parity in 2015 with 48.5% women speakers, along with a dramatic reduction in manels (8). These results demonstrate that it is possible to achieve gender equity and diversity among speakers in a major scientific meeting in a short time frame based on specific awareness made to the leadership and program planning committee.

We recognize that gender equity is not always possible when there are baseline imbalances in the population of experts from which to choose. The finding of disparity of women’s representation in genitourinary cancer topics is in line with the known gender disparity in the field of urology, which appears to extend to the field of urologic oncology (9). A prior analysis of major urology meetings found that, between December 2019 and November 2020, 63.5% of sessions were manels (10), which is notably higher compared with the ASCO Annual Meeting.

Despite the potential for positive change based on presentation of data on manels and gender disparity among invited speakers as demonstrated by the American Society for Microbiology’s experience, it is important to keep in mind that barriers remain (10-15); so much progress is still needed. Although the presence of manels was overall low (and improved) throughout our study period, it is critically important that ASCO leadership and committee membership continue to maintain their progress, specifically in areas where there are obvious gaps. This is particularly important for ASCO Annual Meeting program planning committees, and ASCO as a whole, given the recent data demonstrating that at the 2017 and 2018 ASCO Annual Meetings, men were less likely to introduce women speakers using a professional address (compared with men, 62% vs 81%, *P* < .001) and were often introduced by first name only (17% vs 3%, *P* < .001) (16), demonstrating unconscious bias and reinforcing gender disparities in oncology. Therefore, further improvement of gender parity among invited speakers as well as training and guidelines regarding speaker introductions may help to reduce this bias.

It is also important that panels reflect the demographics of Annual Meeting attendees. In 2017, ASCO queried attendees to collect data on their gender breakdown. Of those who volunteered to categorize their gender as female or male, among full members, 28.5% identified as female vs 28.7% identified as male; among early-career ASCO members, 28.8% identified as female and 33.2% identified as male; and among members-in-training, 23.1% identified as female and 20.7% identified as male (17). Overall, among all 3 groups, 26.6% were female and 27.5% were male attendees. The difference between the 2 genders was less than 1%, reinforcing the need to ensure speaker invitations and panels are reflective of annual meeting attendees. This is also evident when examining all-woman panels, because there should be a balance of gender diversity to gain a range of perspectives and viewpoints. In terms of medical oncology academic faculty, between 2017 and 2019, approximately 36%-38% identified as women (5); of oncology (internal medicine) trainees, between 2018 and 2021, those who identified as women ranged between 20.5% and 33.3%; and of hematology and oncology trainees, those who identified as women ranged between 24.4% and 28.4% (18).

To our knowledge, this is the first evaluation of invited speaker and panel diversity by gender at the one of the largest international oncology meetings over 4 years. There are limitations inherent to the retrospective nature of this study. Because only 1 oncology meeting was studied, there is a chance that these results are not generalizable to the field of oncology as a whole. However, given that this is one of the most widely and globally attended conferences, we feel that these findings are sufficiently representative. In addition, we were limited to only 4 years of data based on data availability at the time of analysis, which may not be sufficient to capture the full range of changes (either positive or negative) over time. However, this analysis represents an initial step and can serve as a benchmark with which to compare future progress. We were not able to capture the age, number of professional years, or educational background of invited speakers and panelists, which could affect our results. Furthermore, we were unable to capture the initial invited speaker(s) by the ASCO committee—it is possible that more women were invited but declined. However, we do note that usually, backup invitees would likely be of the same gender (ie, if an invited woman declined, in general, it is likely that the committee would ask another woman to speak when possible). We also acknowledge that we were unable to deduce race and ethnicity of invited panelists, which is important to evaluate to ensure a diverse representation of views. Finally, we acknowledge that gender is nonbinary, and gender was not self-identified but extracted from various sources; it is possible that these sources were incorrect and speakers or panelists were misclassified. Future analyses using speaker-identified nonbinary gender and race and ethnicity can allow for further insight into the diversity of ASCO-appointed speakers and panels at the ASCO Annual Meeting.

Throughout the evaluated study duration, the number of invited women panelists increased during the study period, with a subsequent decrease in the proportion of manels. We applaud ASCO for striving for gender parity among invited panelists, although there are certain topics or specialties where representation of women has remained stagnant. In addition, the proportion of manels has not improved further from 10% since 2019. Although ASCO planning committees are encouraged to be mindful of the diversity of invited speakers to strive for a balance of viewpoints whenever possible, accountability is necessary to ensure that final panels are reflective of the oncology community. It is imperative that ASCO Leadership and Annual Meeting organizers remain aware of current trends and continue to ensure greater representation of voices amongst invited panelists.

## Supplementary Material

pkad008_Supplementary_Data

## Data Availability

The data underlying this article are accessible via the publicly available ASCO online programs and summarized in the Supplementary Tables (available online).
