# Supplementary material for: Recent trends of “manels”: gender representation among invited panelists at an international oncology conference
Source: JNCI Cancer Spectr. 2023 Feb 10;7(2):pkad008. doi: 10.1093/jncics/pkad008 (PMC9991598; doi:10.1093/jncics/pkad008)
Supplement: pkad008_Supplementary_Data [file pkad008_supplementary_data.pdf]

Supplemental Tables

Supplementary Table 1. Representation of invited women panelists by role, specialty, session type, and topic

	2018-2021			2018		2019		2020		2021		
	**Women**	**Men**	**Total**	**Women**	**Total**	**Women**	**Total**	**Women**	**Total**	**Women**	**Total**	*p-value*
**Role**	**n (%)**	**n (%)**	**N**	**n (%)**	**N**	**n (%)**	**N**	**n (%)**	**N**	**n (%)**	**N**	*time trend*
Panelists*	1181 (47.7)	1294 (52.3)	2475	306 (41.6)	736	347 (48.1)	721	245 (49.6)	494	283 (54.0)	524	<0.001
Chairs/Moderators**	359 (50.6)	350 (49.4)	709	116 (46.8)	248	123 (52.3)	235	46 (50.5)	91	74 (54.8)	135	0.157
**Specialty**	**2018-2021**			**2018**		**2019**		**2020**		**2021**		
	**Women**	**Men**	**Total**	**Women**	**Total**	**Women**	**Total**	**Women**	**Total**	**Women**	**Total**	*p-value*
	**n (%)**	**n (%)**	**N**	**n (%)**	**N**	**n (%)**	**N**	**n (%)**	**N**	**n (%)**	**N**	*time trend*
Medical Oncology	762 (46.4)	880 (53.6)	1642	224 (42.3)	530	214 (45.8)	467	157 (47.7)	329	167 (52.8)	316	0.003
Radiation Oncology	62 (45.9)	73 (54.1)	135	14 (31.8)	44	15 (45.5)	33	9 (47.4)	19	24 (61.5)	39	0.008
Surgery/Anesthesiology	118 (49.8)	119 (50.2)	237	28 (45.2)	62	40 (49.4)	81	23 (56.1)	41	27 (50.9)	53	0.428
Medicine/Pediatrics	58 (48.3)	62 (51.7)	120	6 (46.2)	13	17 (41.5)	41	13 (43.3)	30	22 (61.1)	36	0.146
Pathology/Radiology/Dermatology	16 (26.2)	45 (73.8)	61	5 (21.7)	23	4 (30.8)	13	4 (25.0)	16	3 (33.3)	9	0.574
Other	165 (58.9)	115 (41.1)	280	29 (45.3)	64	57 (66.3)	86	39 (66.1)	59	40 (56.3)	71	0.307
p-value	<0.001			0.265		0.111		0.034		0.608		
**Session Type**	**2018-2021**			**2018**		**2019**		**2020**		**2021**		
	**Women**	**Men**	**Total**	**Women**	**Total**	**Women**	**Total**	**Women**	**Total**	**Women**	**Total**	*p-value*
	**n (%)**	**n (%)**	**N**	**n (%)**	**N**	**n (%)**	**N**	**n (%)**	**N**	**n (%)**	**N**	*time trend*
Scientific	440 (50.9)	425 (49.1)	865	122 (48.8)	250	127 (51.6)	246	84 (50.6)	166	107 (52.7)	203	0.463
Leadership/Special	47 (43.1)	62 (56.9)	109	8 (28.6)	28	7 (26.9)	26	13 (48.2)	27	19 (67.9)	28	0.001
Educational	694 (46.2)	807 (53.8)	1501	176 (38.4)	458	213 (47.4)	449	148 (49.2)	301	157 (53.6)	293	<0.001
p-value	0.058			0.010		0.049		0.930		0.327		
**Topic**	**2018-2021**			**2018**		**2019**		**2020**		**2021**		
	**Women**	**Men**	**Total**	**Women**	**Total**	**Women**	**Total**	**Women**	**Total**	**Women**	**Total**	*p-value*
	**n (%)**	**n (%)**	**N**	**n (%)**	**N**	**n (%)**	**N**	**n (%)**	**N**	**n (%)**	**N**	*time trend*
Population sciences	247 (50.5)	242 (49.5)	489	47 (40.5)	116	79 (52.3)	151	53 (59.6)	89	68 (51.1)	133	0.088
Breast/Gynecologic cancers	158 (58.7)	111 (41.3)	269	48 (57.8)	83	43 (53.8)	80	33 (62.3)	53	34 (64.2)	53	0.347
Genitourinary cancers	54 (38.6)	86 (61.4)	140	23 (41.1)	56	10 (32.3)	31	10 (38.5)	26	11 (40.7)	27	0.969
Other cancers	393 (46.2)	457 (53.8)	850	100 (41.0)	244	100 (45.7)	219	82 (42.7)	192	111 (56.9)	195	0.004
Supportive oncology	83 (70.3)	35 (29.7)	118	31 (64.6)	48	24 (82.8)	29	14 (73.7)	19	14 (63.6)	22	0.937
Translational/Pre-clinical	117 (36.7)	202 (63.3)	319	29 (27.4)	106	38 (39.2)	97	27 (44.3)	61	23 (41.8)	55	0.031
Other	129 (44.5)	161 (55.5)	290	28 (33.7)	83	53 (46.5)	114	26 (48.1)	54	22 (56.4)	39	0.016
p-value	<0.001			<0.001		0.001		0.009		0.154		

*including chairs
**including co-chairs/co-moderators
Specialties:
Surgery: Surgery, Gynecology, Otolaryngology, Neurosurgery, General surgery, Plastic surgery
Medicine: Internal medicine, Family medicine, Pulmonary medicine, Gastroenterology, Cardiology, Endocrinology
Radiology: Radiology, Nuclear medicine
Other: Non-clinical, Immunology, Pharmacology, Palliative Care, Psychiatry, Geriatrics, Other, unknown
Topics:
Population sciences: Care delivery & Regulatory policy, Disparities/Health equity , Prevention, Risk reduction, Hereditary cancer, Health services & Quality improvement, Global health, Ethics
Other cancers: Hematologic, Gastrointestinal, Lung, CNS, Head & Neck, Pediatric, Melanoma/Skin, Sarcoma
Translational/Pre-clinical: Developmental Therapeutics, Immunotherapy, Tumor biology, Precision medicine, Cancer genetics
Supportive oncology: Symptoms & Survivorship, Patient & Survivor care, Geriatric
Other: Special sessions, Professional development & Education advances, Clinical trials, Award lecture

Supplementary Table 2. Manel sessions overall, by session type, and topic

	2018-2021		2018		2019		2020		2021		
	**Manels**	**Total**	**Manels**	**Total**	**Manels**	**Total**	**Manels**	**Total**	**Manels**	**Total**	*p-value*
	**n (%)**	**N**	**n (%)**	**N**	**n (%)**	**N**	**n (%)**	**N**	**n (%)**	**N**	*time trend*
**Overall**	81 (12.1)	670	33 (17.4)	190	18 (10.5)	172	15 (9.6)	157	15 (9.9)	151	0.030
**Session Type**	**2018-2021**		**2018**		**2019**		**2020**		**2021**		
	**Manels**	**Total**	**Manels**	**Total**	**Manels**	**Total**	**Manels**	**Total**	**Manels**	**Total**	*p-value*
	**n (%)**	**N**	**n (%)**	**N**	**n (%)**	**N**	**n (%)**	**N**	**n (%)**	**N**	*time trend*
Scientific	8 (4.2)	189	1 (2.1)	47	1 (2.2)	46	3 (6.3)	48	3 (6.3)	48	0.209
Leadership/Special	6 (17.1)	35	2 (25.0)	8	3 (42.9)	7	1 (10.0)	10	0 (0.0)	10	0.057
Educational	67 (15.0)	446	30 (22.2)	135	14 (11.8)	119	11 (11.1)	99	12 (12.9)	93	0.037
p-value	<0.001		0.001		0.007		0.666		0.354		
**Topic**	**2018-2021**		**2018**		**2019**		**2020**		**2021**		
	**Manels**	**Total**	**Manels**	**Total**	**Manels**	**Total**	**Manels**	**Total**	**Manels**	**Total**	*p-value*
	**n (%)**	**N**	**n (%)**	**N**	**n (%)**	**N**	**n (%)**	**N**	**n (%)**	**N**	*time trend*
Population sciences	15 (10.6)	141	5 (16.1)	31	4 (10.0)	40	0 (0.0)	29	6 (14.6)	41	0.714
Breast/Gynecologic cancers	9 (12.0)	75	3 (13.0)	23	3 (15.8)	19	2 (11.8)	17	1 (6.3)	16	0.503
Genitourinary cancers	5 (13.2)	38	1 (6.7)	15	0 (0.0)	7	3 (33.3)	9	1 (14.3)	7	0.219
Other cancers	24 (11.0)	219	10 (16.7)	60	5 (10.4)	48	6 (10.0)	60	3 (5.9)	51	0.077
Supportive oncology	1 (2.8)	36	1 (7.1)	14	0 (0.0)	7	0 (0.0)	7	0 (0.0)	8	0.286
Translational/Pre-clinical	18 (19.6)	92	9 (30.0)	30	4 (15.4)	26	2 (10.5)	19	3 (17.6)	17	0.189
Other	9 (13.0)	69	4 (23.5)	17	2 (8.0)	25	2 (12.5)	16	1 (9.1)	11	0.333
p-value	0.218		0.465		0.908		0.097		0.588		

Population sciences: Care delivery & Regulatory policy, Disparities/Health equity, Prevention, Risk reduction, Hereditary cancer, Health services & Quality improvement, Global health, Ethics
Other cancers: Hematologic, Gastrointestinal, Lung, CNS, Head & Neck, Pediatric, Melanoma/Skin, Sarcoma
Translational/Pre-clinical: Developmental Therapeutics, Immunotherapy, Tumor biology, Precision medicine, Cancer genetics
Supportive oncology: Symptoms & Survivorship, Patient & Survivor care, Geriatric
Other: Special sessions, Professional development & Education advances, Clinical trials, Award lecture
